# Glutamine Metabolism Drives Growth in Advanced Hormone Receptor Positive Breast Cancer

**DOI:** 10.3389/fonc.2019.00686

**Published:** 2019-08-02

**Authors:** Diane M. Demas, Susan Demo, Yassi Fallah, Robert Clarke, Kenneth P. Nephew, Sandra Althouse, George Sandusky, Wei He, Ayesha N. Shajahan-Haq

**Affiliations:** ^1^Department of Oncology, Lombardi Comprehensive Cancer Center, Georgetown University Medical Center, Washington, DC, United States; ^2^Calithera Biosciences, South San Francisco, CA, United States; ^3^Cell, Molecular and Cancer Biology, Medical Sciences, Indiana University School of Medicine, Bloomington, IN, United States; ^4^Department of Biostatistics, Indiana University School of Medicine, Indianapolis, IN, United States; ^5^Department of Pathology and Laboratory Medicine, Indiana University School of Medicine, Indianapolis, IN, United States; ^6^Program in Genetics, Bioinformatics, and Computational Biology, VT BIOTRANS, Virginia Tech, Blacksburg, VA, United States

**Keywords:** breast cancer, endocrine resistance, glutamine metabolism, mTOR, CB-839, everolimus

## Abstract

Dependence on the glutamine pathway is increased in advanced breast cancer cell models and tumors regardless of hormone receptor status or function. While 70% of breast cancers are estrogen receptor positive (ER+) and depend on estrogen signaling for growth, advanced ER+ breast cancers grow independent of estrogen. Cellular changes in amino acids such as glutamine are sensed by the mammalian target of rapamycin (mTOR) complex, mTORC1, which is often deregulated in ER+ advanced breast cancer. Inhibitor of mTOR, such as everolimus, has shown modest clinical activity in ER+ breast cancers when given with an antiestrogen. Here we show that breast cancer cell models that are estrogen independent and antiestrogen resistant are more dependent on glutamine for growth compared with their sensitive parental cell lines. Co-treatment of CB-839, an inhibitor of GLS, an enzyme that converts glutamine to glutamate, and everolimus interrupts the growth of these endocrine resistant xenografts. Using human tumor microarrays, we show that GLS is significantly higher in human breast cancer tumors with increased tumor grade, stage, ER-negative and progesterone receptor (PR) negative status. Moreover, GLS levels were significantly higher in breast tumors from African-American women compared with Caucasian women regardless of ER or PR status. Among patients treated with endocrine therapy, high GLS expression was associated with decreased disease free survival (DFS) from a multivariable model with GLS expression treated as dichotomous. Collectively, these findings suggest a complex biology for glutamine metabolism in driving breast cancer growth. Moreover, targeting GLS and mTOR in advanced breast cancer may be a novel therapeutic approach in advanced ER+ breast cancer.

## Introduction

About 70% of all breast cancers are estrogen receptor positive (ER+) and are treated with antiestrogens such as Tamoxifen (selective estrogen receptor modulator; SERM), Faslodex/fulvestrant/ICI182,780 (selective estrogen receptor downregulator; SERD) or aromatase inhibitors (AI). However, resistance to such endocrine therapies is common and advanced ER+ breast cancer remains an incurable disease (1–4). Increased growth in advanced cancers demands increased uptake of amino acids to provide a sufficient supply of building blocks of cellular proteins. Particularly, glutamine uptake metabolism is increased in many cancer types (5). Excess glutamine can stimulate activity of the serine/threonine kinase mammalian target of rapamycin complex 1 (mTORC1), a master regulator of both cell signaling and metabolic pathways, to promote cell growth and suppress catabolism or autophagy (5, 6). Reprogramming of cellular metabolism in advanced breast cancers aligns with hyper-activation of mTORC1. Currently, the mTORC1 inhibitor everolimus (Afinitor; RAD001) in combination with the steroidal AI exemestane is approved for treating advanced ER+ with HER2-non-overexpressing tumors, a combination that has shown some clinical benefit (7, 8). Negative feedback loops with AKT activation or increased autophagy that can replenish the amino acid supply, are speculated to account for lack of effectiveness of mTORC1 inhibitors. Thus, the complex relationship between glutamine demand and mTORC1 could be a unique targetable connection in advanced cancers (9).

Endocrine resistant breast cancer cells are more dependent on MYC-regulated glutamine uptake compared with sensitive cells. However, level of glutaminase (GLS), a key enzyme that converts glutamine to glutamate, were not different between these endocrine sensitive and resistant cells (10). Here we show that endocrine resistant breast cancer cells are more dependent on glutamine for growth and this pathway is more resilient to inhibition of glutamine transporters such as ASCT2. Combination of everolimus and CB-839, an inhibitor of glutaminase (GLS), attenuates the growth of endocrine resistant human breast cancer xenografts. We also show that GLS protein levels are increased in aggressive human breast tumors and lower DFS for endocrine therapy. Together, our data suggest that glutamine metabolism in advanced ER+ breast cancers is a promising anti-cancer target.

## Materials and Methods

### Cell Culture and Reagents

LCC1 (estrogen independent, Tamoxifen [TAM] and Faslodex/fulvestrant/ICI182,780 [ICI] sensitive) and LCC9 (estrogen independent, ICI resistant and TAM cross-resistant) cells were established as previously described (11, 12). Cells were grown in phenol red-free IMEM (Life Technologies, Grand Island, NY; A10488-01) with 5% charcoal-stripped calf serum (CCS); this media contains 2 mM L-glutamine and ~12 mM glucose. For glutamine-dependency growth assay, DMEM without glucose or glutamine (Life Technologies; catalog # A14430-01) was used supplemented with 5% CCS (10). CB-839 was generously provided by Calithera Biosciences (South San Francisco, CA). Faslodex (Fulvestrant; ICI182,780 (ICI) was obtained from Tocris Bioscience (Ellisville, MO). Everolimus (RAD100) was purchased from Selleck (Houston, TX). All cells were authenticated by DNA fingerprinting and tested regularly for *Mycoplasma* infection. All other chemicals were purchased from Sigma-Aldrich.

### Cell Proliferation and Viability

For determination of cell density, cells were plated in 96-well plates at 5 × 10^3^ cells/well. At 24 h, cells were treated with specified drugs for 48 h (or otherwise indicated). After treatment, media were removed, and plates were stained with a solution containing 0.5% crystal violet and 25% methanol, rinsed, dried overnight, and re-suspended in citrate buffer (0.1 M sodium citrate in 50% ethanol). Intensity of staining, assessed at 570 nm and quantified using a V_Max_ kinetic microplate reader (Molecular Devices Corp., Menlo Park, CA), is directly proportional to cell number (10).

### Orthotopic Xenografts in Athymic Mice

Five-week-old ovariectomized NCr nu/nu athymic nude mice (Taconic Biosciences, Rensselaer, NY) were injected orthotopically with 1.0 × 10^6^ LCC1/LCC9 cells in 50% Matrigel into mammary fat pads. There were two tumors per mouse and five mice per treatment for each cell line that resulted in ten tumors per treatment group. Treatments were: vehicle alone (for CB-839, 25% hydroxypropyl-β-cyclodextrin in 10 mM citrate, pH 2 (27), or for everolimus, 30% propylene glycol and 5% Tween 80), CB-839 (200 mg/kg by oral gavage twice daily), everolimus (5 mg/kg by intraperitoneal, IP, one injection daily) or the combination of CB-839 and everolimus. Body weight and tumor size were monitored weekly. For all groups, treatment began on day-14 post-inoculation and continued for 3 weeks. All mice were sacrificed at day-35 post-inoculation and tumors were collected for further analysis. Mice were housed and maintained under specific pathogen-free conditions and used in accordance with institutional guidelines approved by Georgetown University Animal Care and Use Committee (GUACUC; protocol #2016-1250).

### Transfections With siRNA

Cells were plated at 60–80% confluence. ASCT2 (10 nM of 3 unique 27mer siRNA duplexes; Origene, Rockville, MD):

SR321780A—rArUrGrUrCrCrCrCrArArCrUrCrArArGrGrCrUrArGrArAAA;
SR321780B—rGrArGrCrCrUrGrArGrUrUrGrArUrArCrArArGrUrGrArAGA;
SR321780C—rCrArArGrCrArCrArUrCrArGrCrCrGrUrUrUrCrArUrCrCTG;

or the control siRNA (universal scrambled negative control; SR30004), were transfected into cells using the RNAiMAX (Invitrogen) transfection reagent. Cells were lysed at 72 h post-transfection and subjected to western blot analysis (below) or the cell density assay (above). Protein levels for ASCT2 relative to actin were quantified using Image J (NIH, USA) (13).

### Western Blot Analysis

Total protein (~20 μg) was isolated from cells following 72 h treatment or vehicle control (0.02% DMSO or ethanol) for protein analysis as previously described (10, 14). The following antibodies were used: ASCT2 (#5345), SNAT1/SLC38A1 (#36057), EAAT2/SLC1A2 (#3838), LAT1/SLC7A5 (#5347), phospho-p70S6K (T389) (#9234), p70SK (#9202), phospho-mTOR(S2448) (#5536), phospho-mTOR(S2481) (#2974), mTOR (#2983), phospho-AKT(S473) (#4058), AKT (#4691), ATG13 (#13273) were from Cell Signaling, Danvers, MA; SNAT2/SLCA2 (#bs-12125R) was from Bioss, Woburn, MA; phospho-ATG13(S318) (#NBP2-19127) was from Novus, Centennial, CO; loading control antibodies such as actin (#sc-47778) was from Santa Cruz Biotechnology, Santa Cruz, CA and β-tubulin (#T7816) was from Sigma.

### Relative Metabolite Quantitation

Targeted mass spectrometry based quantification of glutamine and glutamate from vehicle (DMSO) and CB-839 (500 nM) treated LCC1 and LCC9 cells (72 h) were performed on a Acquity UPLC (Waters Corporation, USA) online with a triple quadrupole MS (Xevo TQ-S Waters Corporation, USA) operating in the MRM mode. For sample preparation, cell pellets were resuspended in 150 μL water and sonicated. Subsequently, 300 μL ACN:MeOH (1:1) containing IS (D5-Glu, 13C5-Gln, (IS conc = 3 μg/mL) was added and the suspension was vortexed and incubated on ice for 15 min and transferred to −20°C overnight. Sample tubes were vortexed and centrifuged at 13,000 *g* for 15 min at 4°C. The supernatant was transferred to a fresh vial, dried under vacuum and re-suspended in 100 μL of CH3OH + water (50:50) prior to transfer to a MS vial. Five microliter was injected on BEH-Amide, 1.7 μm × 2.1 × 100 mm column (Waters Corporation, USA) for LC/MS analysis. Concentrations of the metabolites in each sample were extrapolated from standard curves and normalized to total protein concentration and to the peak area of the internal standards (15).

### Patient Information, Tumor Micro Array (TMA), and Immunohistochemical (IHC) Staining

The TMA was prepared as part of a retrospective study at a central laboratory as the Breast Cancer Tissue Microarray Project: Retrospective Data Collection, IRB Number: NS0910-04 at the University of Indiana (with Vancouver General Hospital). Samples on the TMA were obtained from Indiana University School of Medicine following Institutional Review Board approval (archival cases at Vancouver General Hospital between 1974 and 1995). TMA consisting of duplicate cores of tumors from 292 patients were analyzed for GLS protein expression by IHC. Clinical information including tumor pathology and TMA preparation have been described previously (16). Tumor protein levels of GLS were analyzed by IHC staining using monoclonal antibodies to GLS (#ab15687, Abcam, Cambridge, MA) at the Histopathology and Tissue Shared Resource at Georgetown University Medical Center.

### Cancer Metabolism Gene Expression

Gene expression levels were measured in total RNA isolated from LCC1 and LCC9 xenografts from three different tumors treated with vehicle, CB-839, everolimus or the combination (total = 24 samples), using the Cancer Metabolism panel for the NanoString nCounter platform (Seattle, WA) (17). This platform was selected because it ensures technical reproducibility and allows direct measurement of RNA without enzymatic reactions. Digital transcript counts from the NanoString nCounter assay were normalized using positive control and housekeeping genes following manufacturer's guidelines. Digital transcripts counts were first normalized to the all six positive controls. The geometric mean $\left(\sqrt[{6}]{x_{pos1}{x_{pos2}}^{...}x_{pos6}}\right)$ of positive controls was calculated for each sample and the average of these geometric means was calculated (18). The scaling factor for each sample is the geometric mean of the sample divided by the average. After normalized to the positive controls, the digital counts were normalized to housekeeping genes. Eight housekeeping genes (COG7, EDC3, HDAC3, MTMR14, NUBP1, SF3A3, TLK2, and ZC3H14) were selected; and the normalized procedure was the same as the normalization of the positive controls.

### Statistical Analysis

For TMAs, for each tumor, since duplicate samples were available for each tumor, only the sample with the highest GLS staining (H-score) was included in the analysis. Two hundred ninety-two patients (80%) had GLS values available/readable. Wilcoxon Rank Sum and Kruskal-Wallis tests were used to determine if GLS H-scores were correlated with other tumor markers. Cox proportional hazards regression models were used to determine whether GLS H-scores were related to overall survival (OS; time from surgery to death or censoring) and disease-free survival (DFS; time from surgery to first recurrence or censoring) in multivariable models. In these analyses, GLS H-scores were divided into low and high categories for OS and DFS based on cutoff values that were determined by using the maximum chi-square value for all score values between the 25th and 75th percentile. Multivariable models with the H-score as dichotomous were fit including variables that were significant from the univariable models. Analyses were conducted using SAS Version 9.4. An α level of 5% was used to determine statistical significance. For all other experiments, Statistical analyses were performed using the Sigmastat software package (Jandel Scientific, SPSS, Chicago, IL). Where appropriate, relative cellular metabolites, protein expression and cell proliferation were compared using either a Student's *t*-test or ANOVA with a *post-hoc t*-test for multiple comparisons. Differences were considered significant at *p* ≤ 0.05. The nature of interaction between CB-839 and everolimus in LCC1 or LCC9 cells was defined by the R Index (RI). The RI values were obtained by calculating the expected cell survival (*S*_exp_; the product of survival obtained with drug A alone and the survival obtained with drug B alone) and dividing *S*_exp_ by the observed cell survival in the presence of both drugs (*S*_obs_). *S*_exp_/*S*_obs_ > 1.0 indicates a synergistic interaction (19). In addition, the SynergyFinder R package was used to determine scores for the Highest Single Agent model (HSA) for CB-839 and everolimus in LCC1 and LCC9 cells. A score >10 indicates a synergistic interaction (20).

## Results

### Endocrine Resistant Breast Cancer Cells Show a Deregulated Dependency on Amino Acids

**Figure 1:**
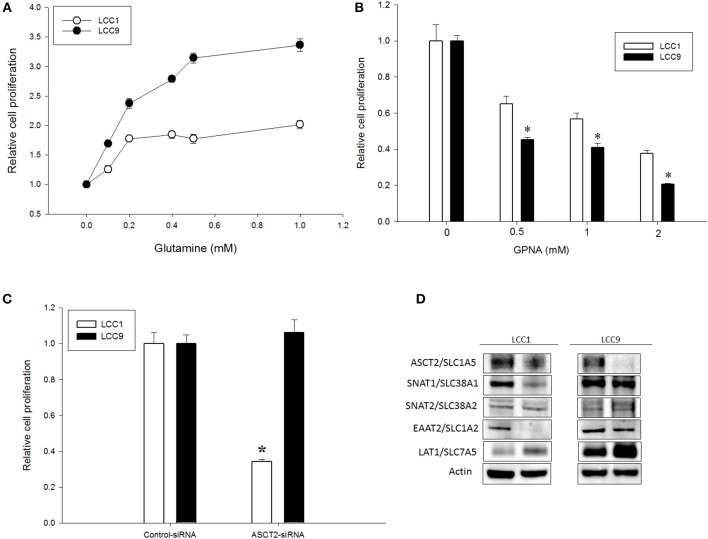
Glutamine dependency is increased but ASCT2 is dispensable in antiestrogen resistant breast cancer cells. **(A)** Glutamine significantly (ANOVA, *p* < 0.01) increased cell proliferation in LCC9 cell compared with LCC1 cells in a dose-dependent manner. Changes in cell proliferation were determined by normalizing cell number measurements at different doses to 0 mM glutamine (vehicle was water). **(B)** LCC9 cells were significantly more sensitive to L-γ-Glutamyl-p-nitroanilide (GPNA), an inhibitor of ASCT2 (SLC1A5) and other sodium-dependent amino acid transporters. Bars represent the mean ± SE of relative number (normalized to vehicle control) for a single representative experiment performed in sextuplicate. All experiments were repeated three times. ANOVA, *p* < 0.001; *p < 0.05 for LCC9 vs. LCC1 for indicated concentrations. **(C)** Knockdown of ASCT2 levels with siRNA in LCC1 cells showed significant decrease in cell number at 72 h compared with that in LCC9 cells. ANOVA, *p* = 0.05; *p ≤ 0.01 for LCC1 ASCT2-siRNA compared with LCC1 control-siRNA. **(D)** Western blotting showed decreased levels of ASCT2 protein in both cell lines following knockdown with ASCT2-siRNA. In LCC1 cells, protein levels of SNAT1 and EAAT2 were decreased while LAT1 was increased with ASCT2 knockdown. In LCC9 cells, SNAT1 and EAAT2 levels were unchanged while LAT1 levels were increased with ASCT2 knockdown; actin was used as a protein loading control.

Increased glutamine demand has been reported in multiple cancers relative to normal tissue (21, 22). In ER+ MCF7-derived antiestrogen resistant LCC9 cells, glutamine metabolism is significantly increased compared with parental antiestrogen sensitive LCC1 cells (10). To determine whether exogenous glutamine differentially affected cell proliferation in these cell lines, we plated LCC1 and LCC9 cells in their regular media (that contains ~2 mM glutamine) for 24 h and then switched to media with 0–1 mM glutamine for another 72 h. Figure 1A shows that cell numbers at 72 h in 0.1, 0.2, 0.4, 0.5, and 1 mM glutamine in LCC9 cells were significantly higher compared with those in LCC1 cells. Recently, ASCT2 (SLC1A5), a sodium-dependent neutral amino acid transporter, which transports glutamine, has been shown to be up-regulated in triple-negative breast cancer cells lines (23). Inhibition of ASCT2 with L-γ-Glutamyl-p-nitroanilide (GPNA), a known inhibitor of ASCT2 (24), showed significant decrease in cell proliferation in LCC9 cells compared to LCC1 cells (Figure 1B). GPNA can inhibit other sodium-dependent amino acid transporters (24). Thus, we knocked down ASCT2 with siRNA to confirm the role of ASCT2 in cell proliferation. Compared with control siRNA, transfection with ASCT2 siRNA resulted in a 20 and 50% reduction in ASCT2 proteins levels in LCC1 and LCC9 cells, respectively (Figure S1). However, cell proliferation was significantly decreased with ASCT2 siRNA compared with control siRNA in LCC1 but not in LCC9 cells (Figure 1C). Down-regulation of ASCT2 decreased levels of other glutamine transporters such as SNAT1 (SLC38A1), SNAT2 (SLC38A2) (25) or glutamate transporters such as EAAT2 (SLC1A2) (26) in LCC1 cells but not in LCC9 cells. ASCT2 knockdown increased LAT1 (SLC7A5), which transports large neutral amino acids including leucine, in both cell lines (Figure 1D). Collectively, these results show that amino acid uptake may be regulated differently in endocrine sensitive and resistant breast cancer cells. Moreover, since cell proliferation was not affected by ASCT2 knockdown in LCC9 cells, the role of ASCT2 is possibly dispensable in endocrine resistant breast cancer cells.

### Inhibitors of Glutaminase and mTOR Synergize to Impede Growth in Endocrine Resistant Breast Cancer Cells and Tumors

**Figure 2:**
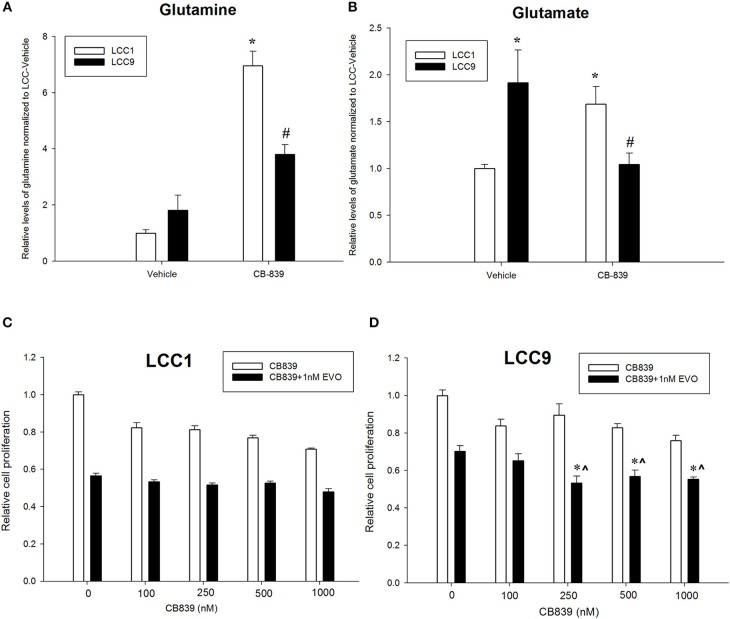
Antiestrogen resistant breast cancer cells show increased sensitivity to anti-glutaminase CB-839. Relative quantification of **(A)** glutamine and **(B)** glutamate in LCC1 and LCC9 cells (*n* = 3 for each treatment per cell line) following treatment with 500 nM CB-839 for 72 h. Treatment with CB-839 significantly (*, ^#^*p* < 0.01) increased glutamine levels in both LCC1 and LCC9 cells compared with vehicle in respective cells. Glutamate levels were significantly higher in LCC9 cells compared with LCC1 cells with vehicle treatment (*p = 0.015). In LCC9 cells, CB-839 significantly decreased (^#^*p* = 0.018) glutamate levels compared with vehicle. Following treatment with CB-839, glutamate levels were significantly (*p = 0.048) increased in LCC1 compared with vehicle. LCC1 **(C)** and LCC9 **(D)** cells were treated with increasing concentrations of CB-839 as indicated in presence of vehicle alone or 1nM everolimus. Bars, ±SE of relative cell numbers (normalized to vehicle control) for a representative experiment performed in sextuplet. Combination with 1 nM everolimus significantly changed the effect of CB-839 on cell proliferation in LCC9 (ANOVA, *p* < 0.001; *p < 0.05 for 1 nM everolimus+CB839 at indicated concentrations vs. 1 nM alone; ^∧^*p* < 0.05 1 nM everolimus+CB-839 vs. CB-839 alone at indicated concentrations. In LCC9 cells, CB-839 synergized with everolimus at 250 and 500 nM with R Index (RI) = 1.1 and 1.02, respectively; RI > 1.0 indicates a synergistic interaction. Furthermore, in LCC9 cells, the Highest Single Agent (HAS) score in combination with 1 nM everolimus, showed a synergistic effect with 250 nM, 500 nM and 1 μM CB-839 (HSA score = 16.8, 13.4, and 14.9, respectively; HSA score >10 indicates synergy.

CB-839 is a potent, selective, and orally bioavailable inhibitor of GLS that have shown anti-tumor properties in ER-independent triple-negative breast cancer (TNBC) (27). To confirm whether metabolism of glutamine was differentially regulated in LCC1 and LCC9 cells, we measured relative levels of metabolites in the glutamine pathway in cells treated with 500 nM CB-839 for 72 h by mass spectrometry. Relative quantification of endogenous levels of glutamine and glutamate were determined in LCC9 and LCC1 cells that were treated with 500 nM CB-839 or vehicle alone for 72 h. In both cell lines, CB-839 treatment significantly (*p* < 0.01) increased the intercellular concentrations of glutamine compared with vehicle controls (Figure 2A). Basal glutamate levels were significantly higher (*p* < 0.05) in LCC9 cells compared with LCC1 cells in the respective vehicle alone groups (Figure 2B). Following treatment with CB-839, glutamate levels in LCC9 significantly decreased (*p* < 0.05) compared with vehicle. Interestingly, in LCC1 cells, CB-839 treatment significantly increased (*p* < 0.05) glutamate levels in LCC1 cells compared with vehicle. Thus, glutamate levels in endocrine resistant LCC9 cells may be more sensitive to inhibition of GLS function by CB-839.

**Figure 3:**
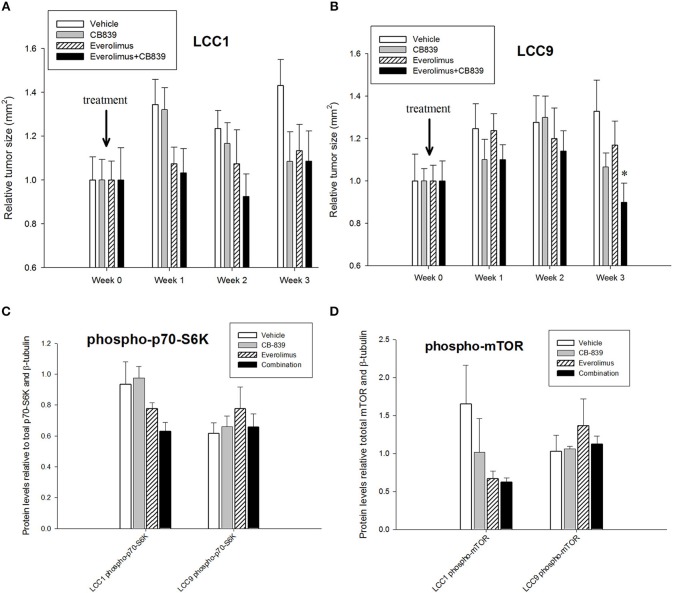
CB-839 and everolimus co-treatment inhibited growth of antiestrogen resistant tumors. Five-week-old ovariectomized athymic nude mice were injected orthotopically with **(A)** LCC1 or **(B)** LCC9 cells and treated with vehicle alone, CB839 (200 mg/kg; twice daily), everolimus (5 mg/kg; once daily) or the combination for 3 weeks. In LCC9 xenografts treated with the combination, tumor growth was significantly (ANOVA; *p* < 0.01) reduced compared to control at week 3. Western blotting of total proteins from xenografts (*n* = 8 tumors per treatment for each cell line) were analyzed for **(C)** phosphorylated mTOR (phosphorylated at S2448 by the PI3K/Akt pathway) and **(D)** p70SK (phosphorylated at T389 by mTOR) to evaluate activation of the mTOR pathway. Phosphorylated p70SK or mTOR was decreased (not significant) in LCC1 tumors treated with everolimus or the combination but this trend was not present in LCC9 xenografts with CB-839, everolimus or the combination compared with vehicle.

Patients with advanced endocrine resistant breast cancer are often treated with an inhibitor of mTOR such as everolimus, along with an aromatase inhibitor such as exemestane. However, clinically meaningful PFS has been modest (28). Since increased glutamine metabolism has been implicated as a compensatory mechanism that contributes to resistance to mTOR inhibition (29), we tested the efficacy of CB-839 as a single agent or in combination with everolimus. CB-839 has a modest effect on both LCC1 (Figure 2C) and LCC9 cells (Figure 2D) cells as a single agent. Combination with 1 nM everolimus significantly changed the effect of CB-839 on cell proliferation in LCC9 (ANOVA, *p* < 0.001; ^*^p < 0.05 for 1 nM everolimus+CB839 at indicated concentrations vs. 1 nM alone; ∧*p* < 0.05 1 nM everolimus+CB-839 vs. CB-839 alone at indicated concentrations. However, there was a synergistic effect only in LCC9 cells which was detected with 250 nM and 500 nM CB-839 (R Index, RI = 1.1 and 1.02, respectively; RI > 1 indicates synergy; see Materials and Methods) (19), respectively, and significantly (*p* < 0.05) inhibited cell proliferation at these concentrations compared with everolimus alone. In addition, the SynergyFinder R package was used to determine scores for the Highest Single Agent (HSA) model to determine nature of interaction for CB-839 and everolimus in the two cell lines. In LCC9 cells, in combination with 1 nM everolimus, there was a synergistic effect with 250, 500 nM, and 1 μM CB-839 (HSA score = 16.8, 13.4, and 14.9, respectively; HSA score>10 indicates synergy; see Materials and Methods) (20). Both RI value and HAS scores for CB-839 and everolimus in LCC1 cells showed additive interactions. Since the metabolic demands of cancer cells *in vitro* can be different from those *in vivo*, due to the absence of the microenvironment and inter-cellular interactions (30), we studied the effect of combining CB-839 and everolimus on tumor size in LCC1 and LCC9 xenografts. NCr nu/nu athymic female nude mice were inoculated with either LCC1 or LCC9 cells. Each cell line group had four treatment arms with 5 mice with two tumors per mouse resulting in 10 tumors each: vehicle alone, CB-839 (200 mg/kg), everolimus (5 mg/kg) or the combination of CB-839 and everolimus (Figures 3A,B). CB-839 was administered by oral gavage twice daily and everolimus was administered by intraperitoneal (IP) injection. In LCC1 xenografts, at week 3 of treatment, CB-839 or everolimus alone, or the combination similarly inhibited the growth of tumors compared with vehicle treatment alone. In comparison, in LCC9 xenografts, at week 3 of treatment with combination of CB-839 and everolimus, tumor growth was inhibited (*p* < 0.01) compared with vehicle alone, while treatment with CB-839 or everolimus alone did not show significant inhibition of tumor growth. Body weight (BW) of each mouse was monitored and no significant change in overall BW was observed (Figure S2).

Western blotting of total proteins from eight tumors per group were analyzed for phosphorylated mTOR (phosphorylated at S2448 by the PI3K/Akt pathway) and p70SK (phosphorylated at T389 by mTOR) to evaluate activation of the mTOR pathway (31). In clinical trials with everoliumus, patients with breast tumors showing high pS6K expression by IHC showed the greatest benefit for time-to-progression (32). Also, since activation of mTORC1 can increase pS6K (33), it has been speculated, but not proven in independent clinical trials, that everolimus may improve outcomes in patients with higher basal expression of these downstream mTORC1 effectors (7). In LCC1 xenografts, there was a trend toward decreased phosphorylated p70SK(T389) or mTOR(S2448) in LCC1 tumors treated with everolimus or the combination (Figure 3C; Figure S3A), although not significantly different. However, this trend was not present in LCC9 xenografts with CB-839, everolimus or the combination compared with vehicle (Figure 3D; Figure S3A). Additionally, we conducted a NanoString analysis of cancer metabolism related genes for each group. Tables 1, 2 show the differentially expressed genes were significantly changed in in LCC1 and LCC9 tumors (n = 3) with CB-839, everolimus or their combination treatment compared with vehicle alone. Based on this gene expression results, we compared the protein levels for TSC2 and ODC1 protein levels in LCC1 and LCC9 tumors, since these genes were significantly changed with CB-839 and everolimus co-treatment in LCC9 tumors compared with vehicle treatment (Figures S3A,B). However, no difference in TSC2 protein levels were observed in LCC9 tumors that were treated with combination of the drugs compared with vehicle alone, and levels of ODC1 in this group were not different (Figures S3C,D). While mTORC1 regulates cell growth and translation, mTORC2 regulates actin organization of the actin cytoskeleton and can phosphorylate AKT at S473 (34). Long-term inhibition with rapamycin can modify mTORC2 levels (35). In LCC1 xenografts, mTORC2 activity, as analyzed by levels of phosho-mTOR(S2481) showed an increase with treatment with everolimus or the combination of CB-839 and everolimus (Figures S4A,B) while phospho-ATK(S473), its substrate, levels remained unchanged. However, in LCC9 xenografts, levels of phosho-mTOR(S2481) and phospho-AKT(S473) (Figure S4C) remained unaffected in treatment groups compared to control. Moreover, we analyzed phospho-ATG13(S318) (Figure S4D) levels since mTOR can block autophagy by hyperphosphorylation of ATG13 (36). In LCC1 xenografts, treatment with CB-839, everolimus or the combination decreased phospho-ATG13(S318) levels compared to control, while in LCC9 xenografts, phospho-ATG13(S318) levels were too low for detection. Thus, the mTOR and autophagy pathways are differentially regulated in LCC9 vs. LCC1 xenografts. Collectively, we show that combination of CB-839 and everolimus is effective in inhibiting growth of endocrine resistant tumors. The signaling mechanism that confers sensitivity to this combination treatment is complex *in vivo*.

**Table 1:** Differentially expressed genes in LCC1 tumors in response to vehicle, CB-839, everolimus or the combination.

**Gene**	**Expanded gene name**	**CB-839/Veh**	***p*-value**	**Everolimus/Veh**	***p*-value**	**Combination/Veh**	***p*-value**
PDGFRA	Platelet derived growth factor receptor alpha	1.39	0.021				
H6PD	Hexose-6-phosphate dehydrogenase/glucose 1-dehydrogenase	−1.21	0.019				
PDGFC	Platelet derived growth factor C/VEGFEl			1.44	0.048		
PFKM	Phosphofructokinase, muscle					−1.20	0.058
DLD	Dihydrolipoamide dehydrogenase					−1.20	0.056
IDH3B	Isocitrate dehydrogenase 3 [NAD(+)] beta					−1.22	0.006
ENO3	Enolase 3					−1.23	0.058
CAD	Carbamoyl-phosphate synthetase 2, aspartate transcarbamylase, and dihydroorotase					−1.25	0.045
TP53	Tumor protein 53/p53					−1.29	0.017
DLAT	Dihydrolipoamide S-acetyltransferase					−1.35	0.012
SLC2A1	Solute carrier family 2 member 1/GLUT-1					−1.39	0.016
G6PD	Glucose-6-phosphate dehydrogenase					−1.39	0.040

**Table 2:** Differentially expressed genes in LCC9 tumors in response to vehicle, CB-839, everolimus or the combination.

**Gene**	**Expanded gene name**	**CB-839/Veh**	***p*-value**	**Everolimus/Veh**	***p*-value**	**Combination/Veh**	***p*-value**
EGLN1	Egl-9 family hypoxia inducible factor 1	−1.20	0.056				
ENO3	Enolase 3	−1.20	0.029			−1.22	0.042
PIK3CA	Phosphatidylinositol-4,5-bisphosphate 3-kinase catalytic subunit alpha	−1.20	0.051				
PDP2	Pyruvate dehyrogenase phosphatase catalytic subunit 2	−1.26	0.011				
PKLR	Pyruvate kinase, liver and RBC	−1.43	0.008				
PDK1	Pyruvate dehydrogenase kinase 1	−1.48	0.050				
RAC2	Rho family, small GTP binding protein Rac2	−1.65	0.041				
SLC5A2	Solute carrier family 5 member 2/Na(+)/glucose cotransporter 1/SGLT2	−1.70	0.021			−1.44	0.059
HK3	Hexokinase 3	−1.75	0.041				
MYC	MYC proto-oncogene, BHLH transcription factor			1.81	0.020	1.81	0.008
VEGFA	Vascular endothelial growth factor A			1.42	0.025	1.34	0.029
ACO1	Aconitase 1/IREBP1			1.24	0.016		
ERBB2	Erb-B2 receptor tyrosine kinase 2/HER2			1.23	0.033	1.31	0.040
LDHB	Lactate dehydrogenase B			−2.00	0.043	−1.73	0.020
PDL1	Phospholipase D1					1.57	0.004
JUN	Jun proto-oncogene, AP-1 transcription factor subunit					1.35	0.015
PRKAA2	Protein kinase AMP-activated catalytic subunit alpha 2					1.28	0.021
TSC2	Tuberous sclerosis 2					1.25	0.009
PFKP	Phosphofructokinase, platelet					1.23	0.042
IKBKB	Inhibitor of nuclear factor kappa B kinase subunit beta					1.22	0.050
H6PD	Hexose-6-phosphate dehydrogenase/glucose 1-dehydrogenase					1.21	0.044
PDHB	Pyruvate dehydrogenase (Lipoamide) beta					−1.20	0.011
SHMT1	Serine hydroxymethyltransferase 1					−1.21	0.023
SDHD	Succinate dehydrogenase complex subunit D					−1.22	0.042
ODC1	Ornithine decarboxylase 1					−1.37	0.024
PRKCB	Protein kinase C beta					−2.13	0.028

### GLS Protein Correlates With Advanced Stage in Human Breast Tumors

**Figure 4:**
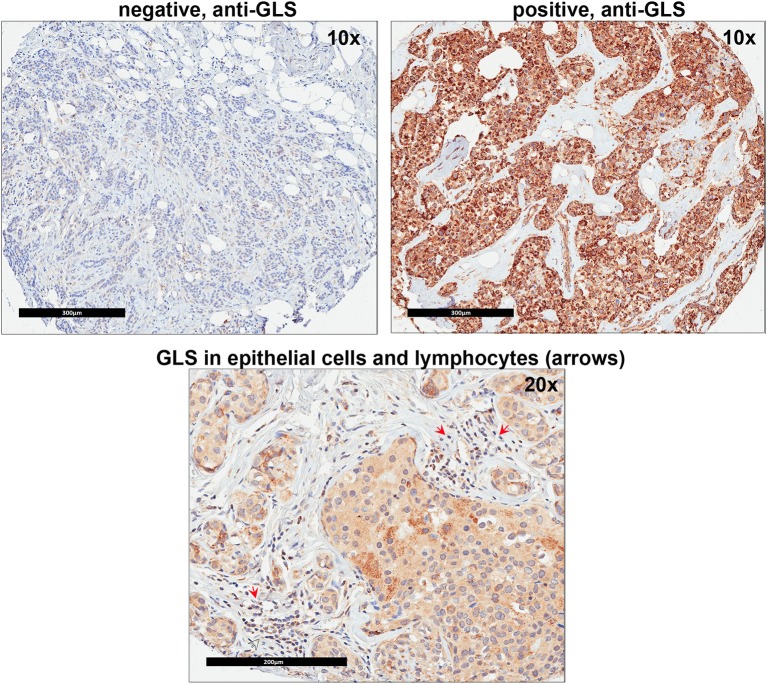
Glutaminase (GLS) protein is increased in advanced breast cancer tumors. A representation of GLS immunohistochemistry staining in tumor microarray (TMA) sample from University of Indiana Simon Cancer Center (see Table 3) is shown. Upper Left Panel, negative control (no primary antibody); Upper Right Panel, positive (with primary antibody) GLS staining was predominantly seen in breast cancer epithelial cells (mainly cytoplasm and nucleus). Lower Panel, in core samples with lymphocytes, GLS staining was also present in lymphocytes (red arrows) along with breast cancer epithelial cells.

Since our data suggests that increased glutamine metabolism drives growth of endocrine resistant breast cancer cells, we measured the protein levels of GLS protein expression in a human breast tumor microarray dataset (Figure 4) that consisted of mostly ER+ tumors; 292 tumors (80%) produced readable data and used in the analysis. The correlations between the GLS H-score with other disease markers are provided in Table 3. GLS levels were found to be correlated with ER and PR status, tumor grade and stage with higher GLS levels in ER-negative, PR-negative and higher tumor grade and stage. Moderate to strong GLS immunostaining was seen in most tumor cells (mainly cytoplasm and nucleus) and the stain was clean with no background except in cases that had lymphocytes in the core along with the tumor cells (Figure 4, lower panel). This pattern of GLS expression was consistent in all arrays with little to no background staining in the other tissues in the core (vascular endothelial cells, smooth muscle cells, fibroblasts, macrophages, and/or scattered lymphocytes infiltrating the tumor region). Considering race within the different breast cancer subtypes, GLS expression was significantly higher (Table 4) in tumors from African-American women compared with those from Caucasian women regardless of ER/PR status. In multivariable analysis, treating GLS H-score as dichotomous, GLS expression was significant for patients treated with endocrine therapy (Table 5) with high GLS expression associated with lower disease-free survival (DFS), however, GLS expression was not significant for overall survival (OS). These findings suggest that tumors with increased GLS levels are aggressive and responded poorly to endocrine therapy.

**Table 3:** Correlations of GLS H-score in a human breast tumor microarray (TMA).

**Variable**	**GLS H-score median (25th percentile, 75th percentile)**						*p*-value*
	***n***	**Values**	***n***	**Values**	***n***	**Values**	
	**Negative**		**Positive**				
ER	65	88.3 (50.5, 120.1)	213	50.1 (26.7, 74.5)			<0.0001
PR	97	75.2 (40.1, 111.6)	167	50.2 (26.0, 74.5)			0.0001
HER-2/neu	120	63.4 (36.4, 89.7)	38	66.2 (24.8, 107.9)			0.8059
ER+/PR+/HER-	53	81.9 (27.7, 112.9)	103	63.1 (34.0, 87.6)			0.1198
Nodal status	166	54.4 (26.2, 89.2)	119	58.0 (34.0, 90.9)			0.2926
	**Caucasian**		**African American**				
Race	230	51.7 (27.7, 87.2)	59	81.9 (42.7, 108.4)			0.0085
	**Grade 1**		**Grade 2**		**Grade 3**		
Tumor grade	67	51.9 (22.7, 71.6)	126	46.5 (24.8, 74.5)	78	89.6 (54.1, 117.9)	<0.0001**
	**T0/1**		**T2**		**T3/4**		
Tumor stage	152	49.9 (26.1, 82.2)	106	58.4 (33.2, 97.5)	33	75.8 (47.6, 110.4)	0.0117**

* From Wilcoxon Rank Sum test for Hormone Receptor Status and Kruskal-Wallis test for Tumor Grade and Tumor Stage.

** *The difference between Grade 1 and 3 was significant (p <0.0001) and Grade 2 and 3 (p <0.0001). The difference between T0/1 and T3/4 was significant (p = 0063)*.

**Table 4:** Correlations for race within different breast cancer subtypes.

**Variable**	**GLS H-score Median (25th percentile, 75th percentile)**				*p*-value*
	***n***	**Values**	***n***	**Values**	
	**Caucasian**		**African American**		
ER+	172	48.9 (26.9, 72.5)	38	63.4 (21.7, 88.7)	0.3107
PR+	139	50.1 (26.7, 73.6)	25	61.7 (21.3, 87.6)	0.9781
Triple Negative	8	93.0 (54.4, 140.8)	7	97.2 (86.2, 132.5)	0.8647

* *Wilcoxon Rank Sum test*.

**Table 5:** Disease Free Survival (DFS) and Overall Survival (OS) with GLS H score as dichotomous variable in multivariable models; *referent group listed second.

**Group**	H-Score category parameter*	***p*-value**	**Point estimate**	**Lower 95% Wald confidence limit**	**Upper 95% Wald confidence limit**
DFS for patients with endocrine therapy	High vs. Low	0.0223	1.934	1.098	3.406
OS for patients with endocrine therapy	High vs. Low	0.1411	0.597	0.301	1.186

## Discussion

Deregulated cellular metabolism is a hallmark of cancer cells (37, 38) and increased glutamine metabolism has been reported in several cancer types (21, 39, 40). Accumulating data suggest that drug resistance in cancer is associated with specific changes in metabolic pathways that favor growth (41–43). In breast cancer, glutamine metabolism is associated with aggressive subtypes (27, 44–46) and antiestrogen resistance (10). The glutamine pathway leads to oxidation at the mitochondria to generate ATP and to synthesis of multiple molecules in the cytosol (5, 22). In this study, we show that endocrine resistant LCC9 breast cancer cells show increased dependence on glutamine compared with parental LCC1 sensitive cells (Figure S5). Several ubiquitous and redundant transporters have been reported for glutamine (39) and some such as ASCT2, ABT0+, and LAT1 are overexpressed in many cancers (47, 48). However, little is known about how glutamine transporters are regulated. Inhibition of ASCT2 significantly reduced cell proliferation in LCC1 cells along with subsequent decrease in levels of glutamine or glutamate transporters such as SNAT1/SLC38A1 and EAAT2/SLC1A2, respectively; these changes were absent in LCC9 cells (Figure 1C). Thus, rewiring of signaling pathways in endocrine resistant cells allows a redundant panel of transporters to maintain glutamine uptake collectively.

Everolimus exerts its inhibitory effects on the mTOR pathway by specifically targeting mTOR complex 1 (mTORC1) without binding to mTOR complex 2 (mTORC2) (7). Inhibition of the mTOR pathway constrains cell growth and proliferation primarily by inhibiting translation. Based on clinical trials, mTOR inhibition in combination with an endocrine therapy is a new therapeutic strategy for women with advanced breast cancer who have previously relapsed on a non-steroidal aromatase inhibitor (49). Combination of everolimus and an aromatase inhibitor synergistically inhibited proliferation and triggered apoptotic cell death in estrogen-sensitive MCF7 breast cancer cells models (50). The efficacy of everolimus and antiestrogens in endocrine resistant cells remains unclear. Previously, we have shown that the oncoprotein MYC is increased in estrogen independent and antiestrogen resistant breast cancer cells compared with parental MCF7 cells (10). Moreover, glutamine dependence is increased in LCC9 cells compared with LCC1 cells without any changes in total GLS levels. Here we show that treatment with GLS inhibitor CB-839 significantly decreases glutamate and increases glutamine in LCC9 cells (Figure 2B) compared with vehicle treatment. Moreover, in LCC9 cells, combination of CB-839 and everolimus synergistically inhibited cell proliferation *in vitro* (Figure 2D) and prevented growth of xenografts (Figure 3B). Gene expression profile analysis from the NanoString Cancer Metabolism panel showed a significant decrease in ODC1 and an increase in TSC2 mRNA expression in LCC9 xenografts when these were co-treated with CB-839 and everolimus. While not significant, TSC2 proteins levels were increased in LCC9 treated with combination of the drugs compared to vehicle alone (Figure S3). TSC2 is a negative regulator of mTOR and mutations in this gene have been correlated with mTOR activation and an increased response to mTOR inhibitors in tumors (51). Conversely, ODC1 protein levels were decreased (not significantly) in tumors treated with combination of the drugs compared with vehicle alone. Since transcript levels are not always adequate predictors of protein levels (52), other mechanisms may contribute to increased sensitivity to CB-839 plus everolimus including micro-RNA (miRNA) regulation of the ODC1-mediated pathway. ODC1 levels are known to be regulated by glutamine in intestinal cells (53). Whether increased ODC1 levels in LCC9 xenografts treated with CB-839 and everolimus reflect a disruption of the polyamine pathway, which is known to promote breast cancer cells growth (54, 55), remains to be clarified. Low levels of phospho-ATG13 in LCC9 xenografts suggest increased levels of basal autophagy (Figure S4D). LCC9 cells have been previously shown to depend on increased pro-survival autophagy (56), and therefore, it is possible that efficacy of CB-839 and everolimus is due to disruption of amino acid metabolism following catabolism of macromolecules via autophagy.

GLS protein levels in breast cancers patient tumors significantly correlated with increased tumor grade and stage (Table 3) confirming the role of increased glutamine metabolism in aggressive breast cancers. Our findings also showed a correlation between GLS levels and ER and PR, and that it is higher in ER- and PR- tumors. Previously, high stromal GLS levels were reported in HER2+ tumors (44). However, in our TMA samples, GLS staining was present predominantly in cancer epithelial cells and there was no correlation with HER2 expression. GLS levels were reported to increase in TNBC breast cancer cells (27, 46) but the significance of GLS protein levels in TNBC tumors remain to be elucidated. Furthermore, increased glutamate levels were reported to be increased in TNBC tumors compared with ER+ tumors (57), highlighting a specific role of glutamine metabolism in breast tumors that are not dependent on estrogen signaling for growth. Interestingly, in our TMA analysis, GLS expression was significantly higher in tumors from African-American (AA) women regardless of hormone receptor or growth factor status. Previously, a tumor subtype, with high tissue oncometabolite 2-hydroxyglutarate (2HG), irrespective of hormone or growth factor receptor, was associated with stem cell-like transcriptional signature, glutaminase overexpression, poor prognosis and occurred with higher frequency in AA patients (58). High GLS level were significantly associated with decreased DFS but not with OS in patients treated with endocrine therapy. While increased GLS levels may contribute to resistance to endocrine therapy, prospective studies are needed to confirm these findings. Our knowledge of metabolite profile of breast tumor subtypes remains incomplete. Additional research is needed to understand whether this type of profiling aligns with the clinical classification of breast cancers that are based on hormone receptor status.

CB-839 has been tested in Phase 1 clinical trials in multiple solid and hematological cancers (NCT02071888, NCT02071862, NCT02071927, and NCT02771626). More recently, CB-839 is in Phase 2 study of the combination of CB-839 with paclitaxel in patients of African ancestry and non-African ancestry with advanced TNBC (NCT03057600) based on earlier studies that showed increased efficacy of CB-839 in inhibiting growth in TNBC cell lines (27). CB-839 is also being evaluated in Phase 2 study in combination with everolimus in renal cell carcinoma (NCT03163667). To date, the metabolic signature of endocrine resistant breast cancers remains unclear, but glutamine metabolism is likely to be important to sustain this phenotype (10, 59). In summary, our study shows that glutamine pathway is altered in endocrine resistant breast cancer cell models and co-targeting enhanced glutamine requirement with mTOR (Figure S5) may be useful in impeding growth of this advanced stage of ER+ breast cancer. Further studies in multiple models of endocrine resistance and human breast cancer samples are needed to determine whether deregulation of glutamine metabolism is a general phenotype in endocrine resistance.

## Data Availability

The raw data supporting the conclusions of this manuscript will be made available by the authors, without undue reservation, to any qualified researcher.

## Ethics Statement

The TMA was prepared as part of a retrospective study at a central laboratory as the Breast Cancer Tissue Microarray Project: Retrospective Data Collection, IRB Number: NS0910-04 at the University of Indiana (with Vancouver General Hospital).

Mice were housed and maintained under specific pathogen-free conditions and used in accordance with institutional guidelines approved by Georgetown University Animal Care and Use Committee (GUACUC).

## Author Contributions

AS-H, SD, RC, and KN contributed to concept design, planning of the study, revision, and final approval of present article. DD, YF, GS, SA, and WH are responsible for doing the experiments, writing, analysis, interpretation, revision, and final approval of present article. All authors have read and approved the final manuscript.

### Conflict of Interest Statement

AS-H has received a research grant from Calithera Biosciences to partly support this project. SD is employed by Calithera Biosciences and contributed to concept design and revision and final approval of present article. The remaining authors declare that the research was conducted in the absence of any commercial or financial relationships that could be construed as a potential conflict of interest.

## Supplementary Material

Figure S1 ASCT2 protein levels following siRNA knockdown in LCC1 and LCC9 cells. **(A)** Western blot showing ASCT2 protein levels in three independent experiments (experiment #1 is the same sample as shown in Figure 1D) following transfection with control or ASCT2 siRNA for 72 h. Actin was used as the loading control. **(B)** Bars represent the mean±SE of relative ASCT2 protein levels (normalized to actin) for the three experiments. ASCT2 protein levels were 20% and 50% reduced in LCC1 and LCC9 cells, respectively.

Figure S2 Body weight (BW) of mice over time. Total body weight did not vary significantly in mice within the different groups with **(A)** LCC1 or **(B)** LCC9 xenografts over time.

Figure S3 Antiestrogen resistant tumors show changes in ODC1 and TSC2 protein levels following co-treatment with everolimus and CB-839. **(A)** Western blots show protein levels of TSC2, phospho-mTOR(S2448), mTOR, phosphor-p70SK(T389), p70SK, and actin (loading control) in LCC1 (*n* = 10 per treatment group), and LCC9 (*n* = 10 per treatment group except CB-839 where *n* = 8) xenografts from different treatment groups. **(B)** Western blots show protein levels of ODC1 in LCC1 (*n* = 3 per treatment group). Graphical representation of Western blotting analysis of proteins from LCC1 or LCC9 xenografts treated with vehicle, CB-839, everolimus or the combination showing **(C)** TSC2 and **(D)** ODC1 protein levels; increase in protein levels in LCC9 tumors treated co-treated with both CB-839 and everolimus were not significant.

Figure S4 Activation of mTORC2 and autophagy are differentially regulated in antiestrogen resistant tumors. **(A)** Western blots show protein levels of phospho-mTOR(S2481), mTOR, phospho-AKT(S473), AKT, phospho-ATG13(S318), ATG13, and b-tubulin (loading control) in LCC1 (*n* = 3 per treatment group) and LCC9 (*n* = 3 per treatment group except CB-839 where *n* = 8) xenografts from different treatment groups. Graphical representation of Western blotting analysis of proteins from LCC1 or LCC9 xenografts treated with vehicle, CB-839, everolimus or the combination showing **(B)** phospho-mTOR(S2481; mTOR2 function), **(C)** phospho-AKT(S473; AKT activation), and **(D)** phospho-ATG13(S318; inhibition of autophagy). Levels of phospho-mTOR(S2481), phospho-AKT(S473), and phospho-ATG13(S318) changed in LCC1 xenografts following treatment in accordance with increase in mTORC2 function and autophagy. In LCC9 xenografts, phospho-mTOR(S2481), and phospho-AKT(S473) levels remained unchanged with treatment while levels of phospho-ATG13(S318) were very low suggesting the presence of a deregulated mTOR pathway and increased basal autophagy in these cells.

Figure S5 Schematic illustration showing possible benefit of combining CB-839 and Everolimus in inhibiting growth in antiestrogen resistant breast cancers. Increased glutamine uptake and metabolism may be coupled with mTORC1 activation in endocrine resistant ER+ breast cancer cells. CB-839 is a potent, selective, reversible, and orally bioavailable inhibitor of human glutaminase (GLS) that can inhibit cellular glutamine to glutamate metabolism. Everolimus is an inhibitor of mTORC1 signaling that can decrease protein synthesis and cell growth. Since activation of both mTOR signaling and glutamine metabolism pathways can lead to increased cell growth, simultaneous inhibition of both pathways maybe a plausible strategy in impeding growth in antiestrogen resistant breast cancer. Text in black denotes parts of the pathways that are addressed in this study while those in grey are included to highlight adjacent mechanisms. Dashed lines or arrows denotes a multipart relationship.
