# Supplementary figures and images for: Glutamine Metabolism Drives Growth in Advanced Hormone Receptor Positive Breast Cancer
Source: Front Oncol. 2019 Aug 2;9:686. doi: 10.3389/fonc.2019.00686 (PMC6688514; doi:10.3389/fonc.2019.00686)

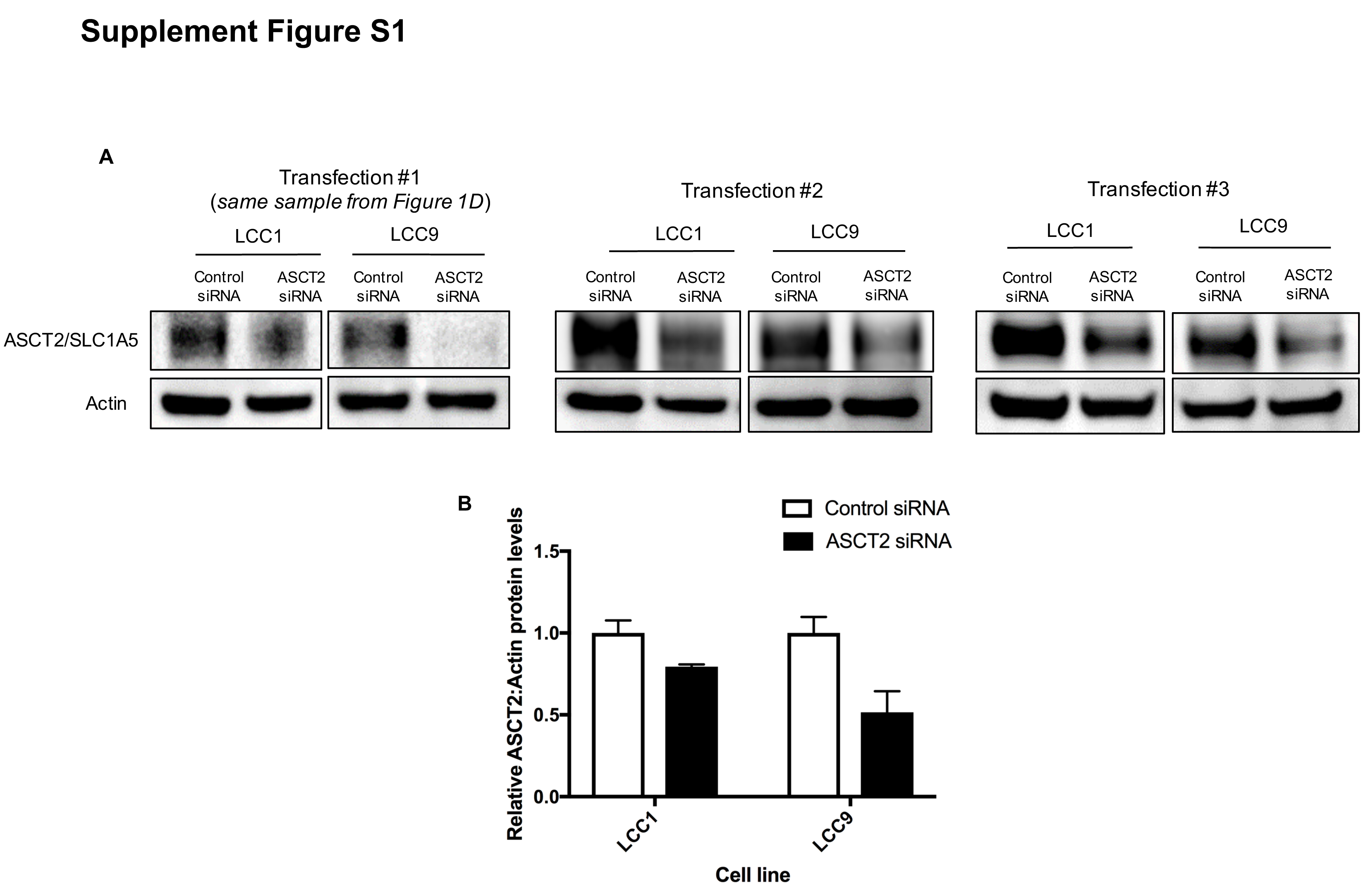

Supplement: Figure S1 — ASCT2 protein levels following siRNA knockdown in LCC1 and LCC9 cells. (A) Western blot showing ASCT2 protein levels in three independent experiments (experiment #1 is the same sample as shown in Figure 1D) following transfection with control or ASCT2 siRNA for 72 h. Actin was used as the loading control. (B) Bars represent the mean±SE of relative ASCT2 protein levels (normalized to actin) for the three experiments. ASCT2 protein levels were 20% and 50% reduced in LCC1 and LCC9 cells, respectively. [file Image_1.TIF]

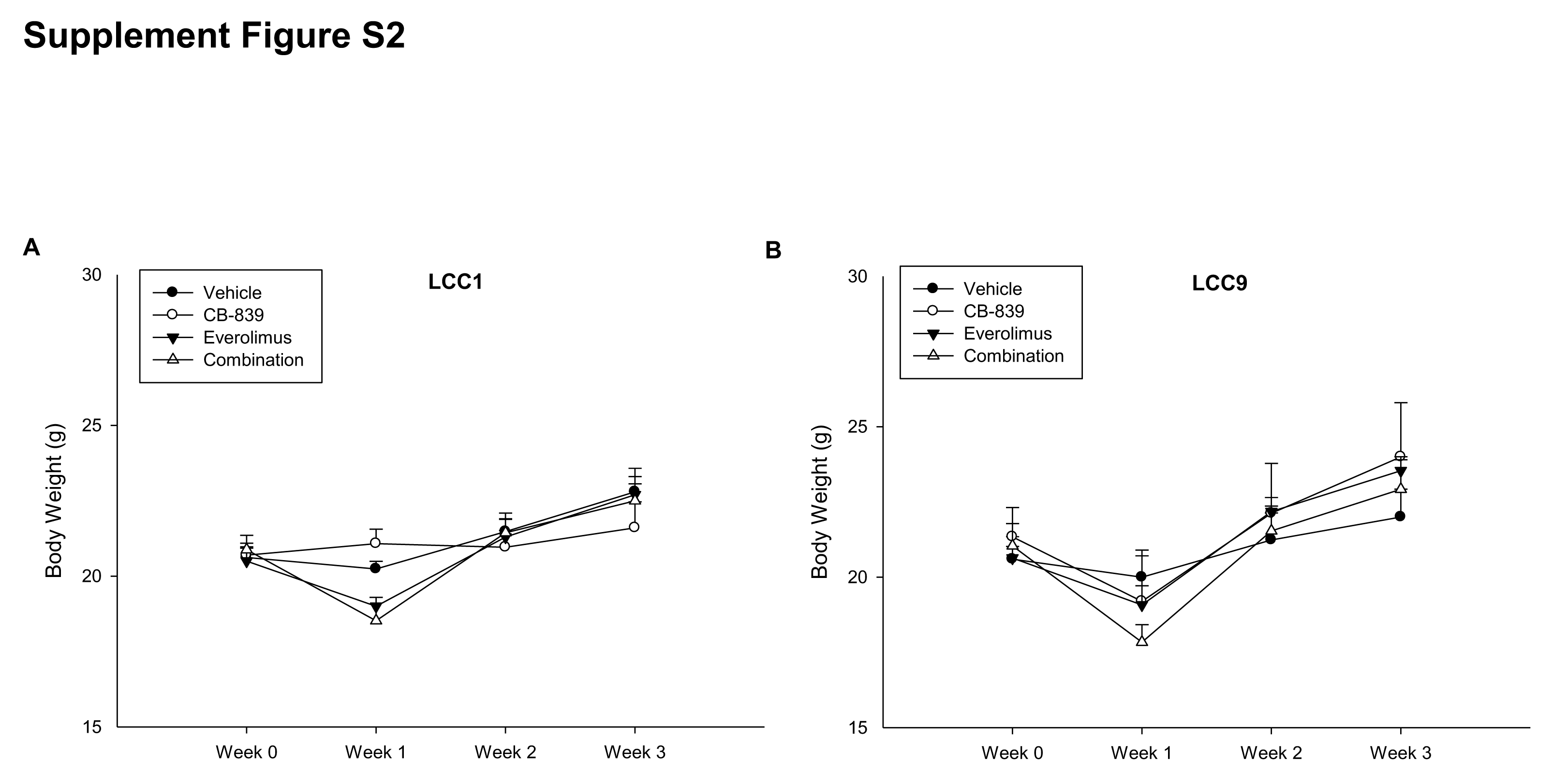

Supplement: Figure S2 — Body weight (BW) of mice over time. Total body weight did not vary significantly in mice within the different groups with (A) LCC1 or (B) LCC9 xenografts over time. [file Image_2.TIF]

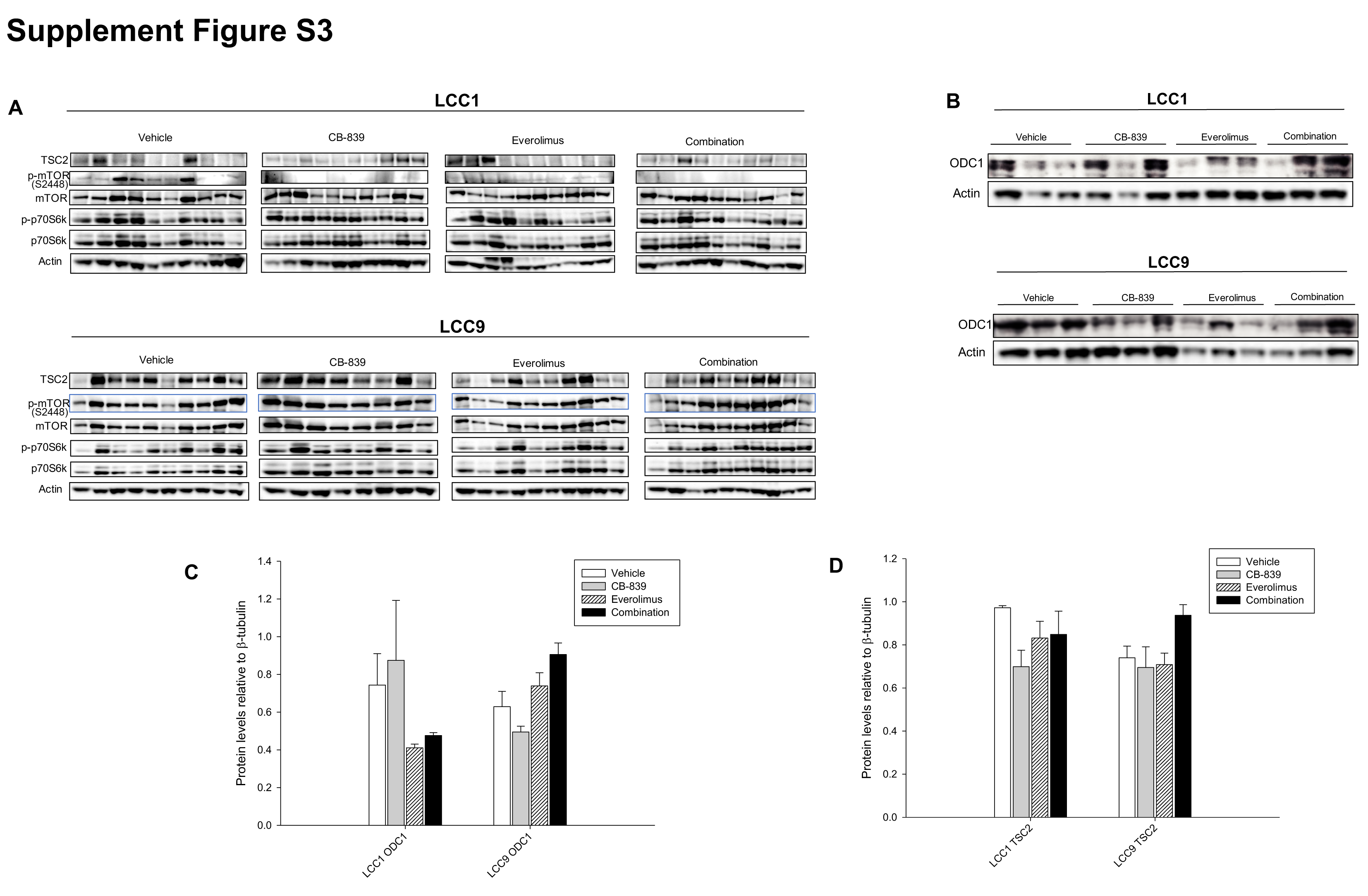

Supplement: Figure S3 — Antiestrogen resistant tumors show changes in ODC1 and TSC2 protein levels following co-treatment with everolimus and CB-839. (A) Western blots show protein levels of TSC2, phospho-mTOR(S2448), mTOR, phosphor-p70SK(T389), p70SK, and actin (loading control) in LCC1 (n = 10 per treatment group), and LCC9 (n = 10 per treatment group except CB-839 where n = 8) xenografts from different treatment groups. (B) Western blots show protein levels of ODC1 in LCC1 (n = 3 per treatment group). Graphical representation of Western blotting analysis of proteins from LCC1 or LCC9 xenografts treated with vehicle, CB-839, everolimus or the combination showing (C) TSC2 and (D) ODC1 protein levels; increase in protein levels in LCC9 tumors treated co-treated with both CB-839 and everolimus were not significant. [file Image_3.TIF]

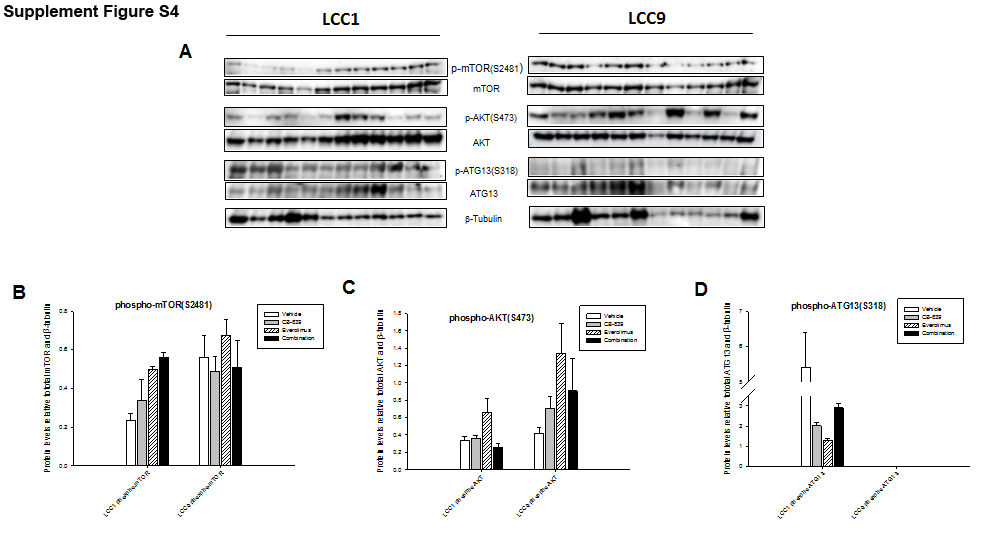

Supplement: Figure S4 — Activation of mTORC2 and autophagy are differentially regulated in antiestrogen resistant tumors. (A) Western blots show protein levels of phospho-mTOR(S2481), mTOR, phospho-AKT(S473), AKT, phospho-ATG13(S318), ATG13, and b-tubulin (loading control) in LCC1 (n = 3 per treatment group) and LCC9 (n = 3 per treatment group except CB-839 where n = 8) xenografts from different treatment groups. Graphical representation of Western blotting analysis of proteins from LCC1 or LCC9 xenografts treated with vehicle, CB-839, everolimus or the combination showing (B) phospho-mTOR(S2481; mTOR2 function), (C) phospho-AKT(S473; AKT activation), and (D) phospho-ATG13(S318; inhibition of autophagy). Levels of phospho-mTOR(S2481), phospho-AKT(S473), and phospho-ATG13(S318) changed in LCC1 xenografts following treatment in accordance with increase in mTORC2 function and autophagy. In LCC9 xenografts, phospho-mTOR(S2481), and phospho-AKT(S473) levels remained unchanged with treatment while levels of phospho-ATG13(S318) were very low suggesting the presence of a deregulated mTOR pathway and increased basal autophagy in these cells. [file Image_4.TIF]

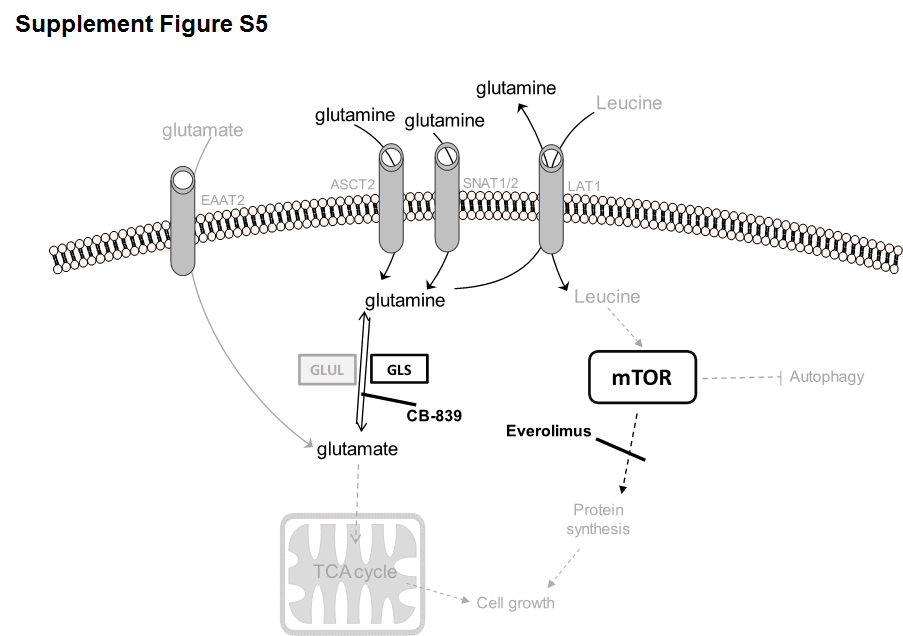

Supplement: Figure S5 — Schematic illustration showing possible benefit of combining CB-839 and Everolimus in inhibiting growth in antiestrogen resistant breast cancers. Increased glutamine uptake and metabolism may be coupled with mTORC1 activation in endocrine resistant ER+ breast cancer cells. CB-839 is a potent, selective, reversible, and orally bioavailable inhibitor of human glutaminase (GLS) that can inhibit cellular glutamine to glutamate metabolism. Everolimus is an inhibitor of mTORC1 signaling that can decrease protein synthesis and cell growth. Since activation of both mTOR signaling and glutamine metabolism pathways can lead to increased cell growth, simultaneous inhibition of both pathways maybe a plausible strategy in impeding growth in antiestrogen resistant breast cancer. Text in black denotes parts of the pathways that are addressed in this study while those in grey are included to highlight adjacent mechanisms. Dashed lines or arrows denotes a multipart relationship. [file Image_5.TIF]
